# A Case of Bronchogenic Cyst Detected by Ultrasound

**DOI:** 10.2174/0115734056347512250219063009

**Published:** 2025-03-14

**Authors:** Lei Zhang, Dong-hui Ji, Kuo-peng Liang

**Affiliations:** 1 Department of Ultrasound, Xingtai People’s Hospital, Hebei, China

**Keywords:** Bronchogenic cyst, Echocardiography, Computed tomography, Congenital anomalies, Intrapulmonary cysts, Chest pain

## Abstract

**Background:**

Bronchogenic cysts are congenital cystic anomalies of the bronchus that originate from abnormal development of the bronchial tree during the embryonic period. Their common manifestation is a space-occupying lesion in the lungs or mediastinum. Common imaging modalities for detecting bronchogenic cysts include chest X-ray and chest computed tomography (CT) scans.

**Case Presentation:**

A 24-year-old female presented with an abnormal space-occupying lesion in the mediastinum detected through imaging examinations. Echocardiography revealed a cystic mass located between the descending aorta and the right pulmonary artery. A CT scan identified a low-density mass with a distinct density relative to adjacent tissues, situated near the left main bronchus. The final diagnosis of a bronchogenic cyst was established following surgical intervention and pathological examination.

**Conclusion:**

Bronchogenic cysts are rare congenital anomalies. Common clinical symptoms include chest pain, cough, and dyspnea. On standard chest radiographs and CT scans, most cysts present as homogenous water-density shadows, with the mediastinum being the most frequently affected location. The diagnosis is confirmed through pathological examination. Surgical intervention remains the most effective treatment method, typically resulting in a favorable prognosis.

## 1 INTRODUCTION

Bronchogenic cysts are rare congenital respiratory anomalies. Typical symptoms include chest pain and cough, with the mediastinum and lungs being the most commonly affected regions. CT is a crucial diagnostic modality, often revealing a round or circular, thin-walled cystic structure with well-defined edges. The cyst may contain liquid, gas, or both. Histological examination commonly identifies characteristic bronchogenic tissues, including bronchial glands, cartilage, elastic fibers, and smooth muscle. The cyst wall is lined by pseudostratified ciliated columnar epithelium or, occasionally, a single layer of squamous epithelium. Surgical interventionremains the primary treatment approach. This report presents a case of a bronchogenic cyst identified through echocardiography, the first such case to the author's knowledge.

## 2 CASE PRESENTATION

A 24-year-old female presented with left-sided chest discomfort and pain, accompanied by a sensation of a foreign body within the chest cavity. The patient sought medical evaluation, which included a detailed review of her medical history and a physical examination. She reported a history of good health.

**Fig. (1A,B):**
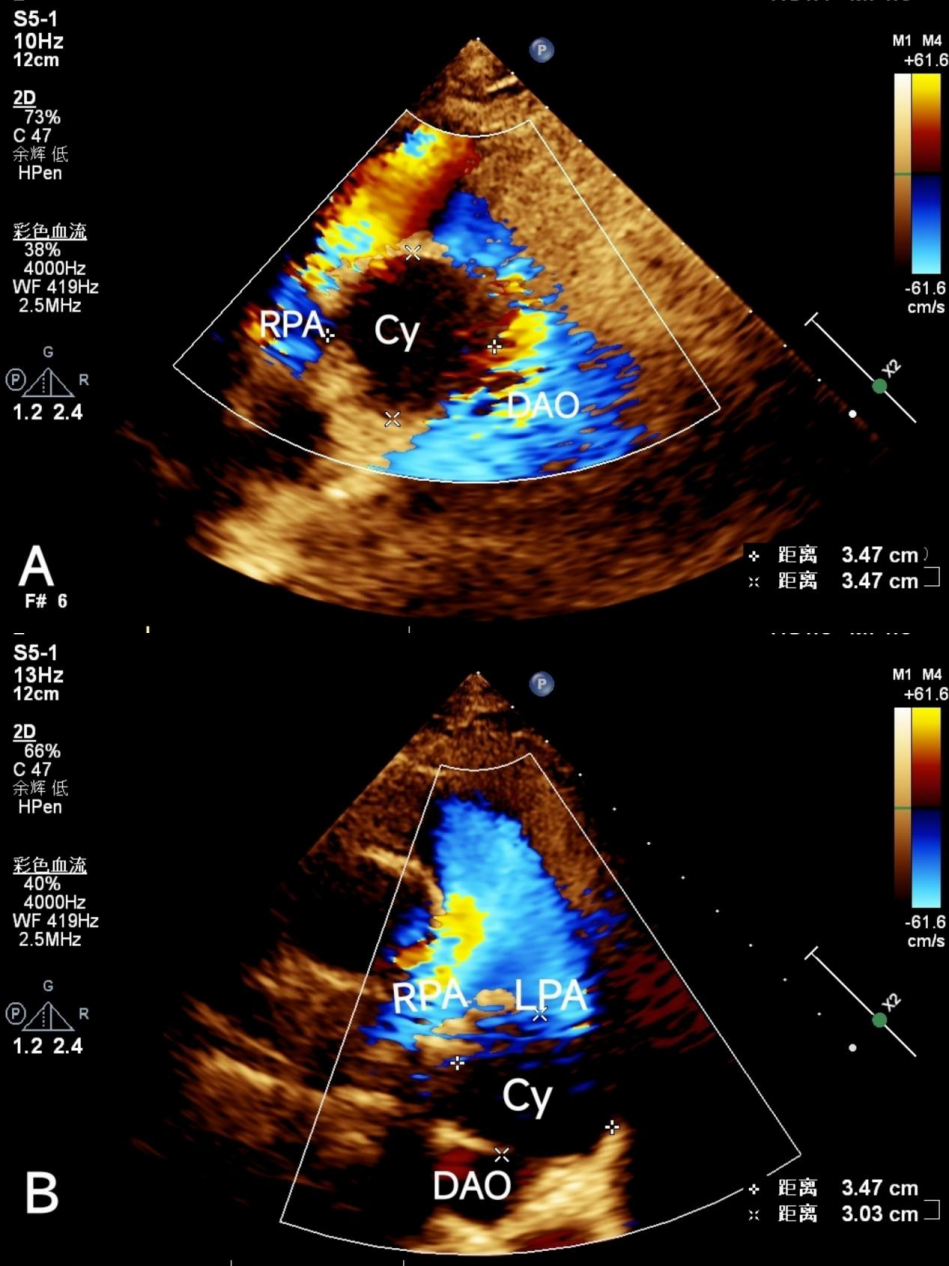
Echocardiographic imaging showing the precise localization of the cyst. (Cy: cyst; RPA: right pulmonary artery; LPA: left pulmonary artery; DAO: descending aorta).

Echocardiography revealed a cystic mass, specifically a fluid-filled sac-like structure, located between the descending aorta and the right pulmonary artery. This finding raised suspicion of a bronchogenic cyst, a benign lesion that typically originates from the lining of the bronchi (Fig. **1**).

**Fig. (2A,B):**
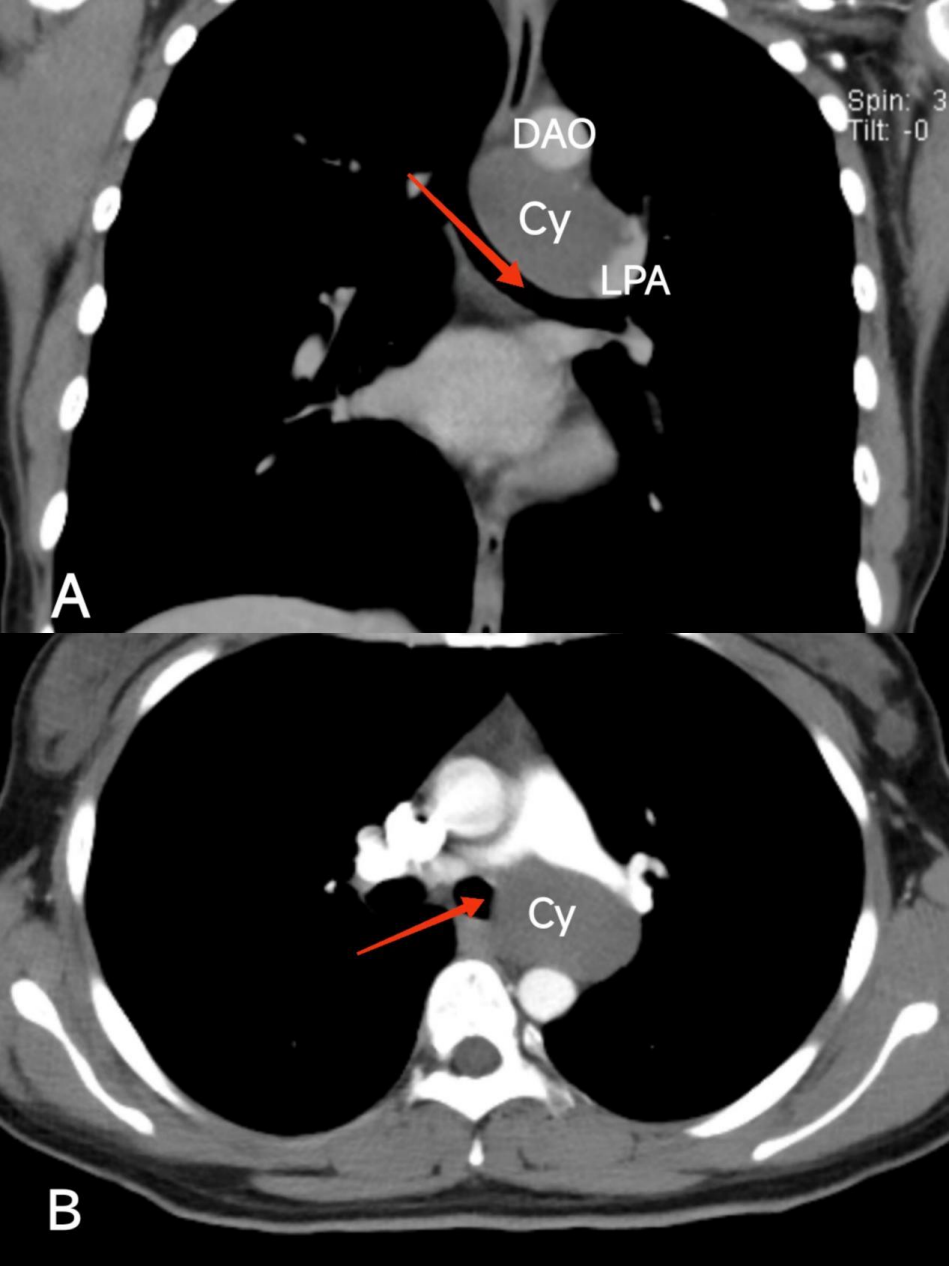
Chest axial and coronal CT scans showing the adjacent relationship and characteristics of the lung cyst. (Cy: cyst, LPA: left pulmonary artery, DAO: descending aorta, red arrow: left main bronchus).

To confirm the diagnosis, a chest-enhanced CT scan was performed. The CT scan identified a low-density mass with a distinct density compared to adjacent tissues, situated near the left main bronchus. This finding confirmed the suspicion of a bronchogenic cyst (Fig. **2**).

**Fig. (3):**
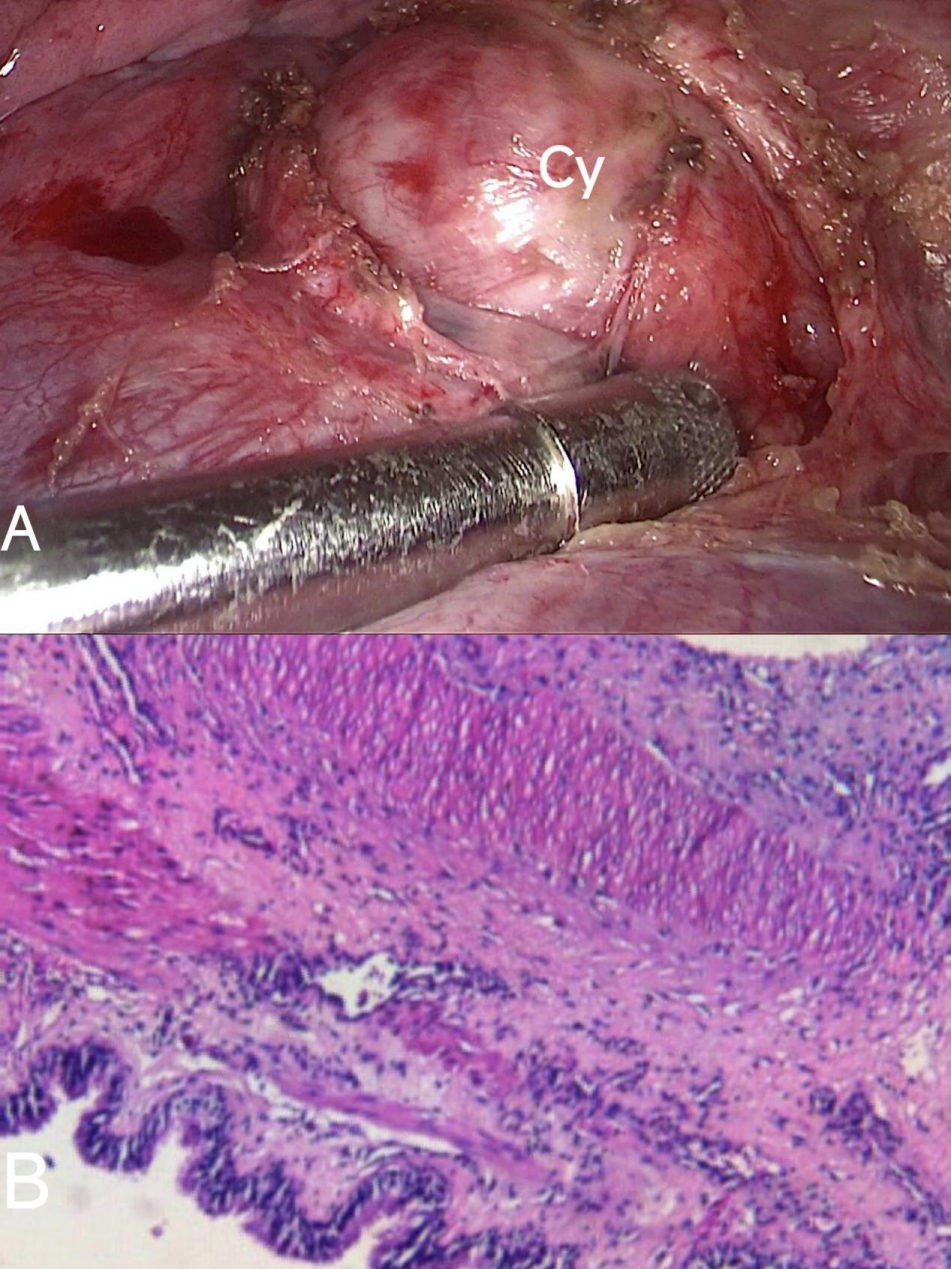
(**A**) Thoracoscopic view of the bronchogenic cyst resection (Cy: cyst). (**B**) Histological findings showing ciliated columnar epithelial cells with serous glands consistent with bronchogenic cysts.

Based on the diagnostic findings, a multidisciplinary medical team concluded surgical intervention to be necessary for both diagnostic confirmation and therapeutic excision. During surgery, pathological examination confirmed the presence of a bronchogenic cyst associated with the left main bronchus (Fig. **3**).

The patient recovered well postoperatively and remained recurrence-free during a one-year follow-up after discharge.

## 3 DISCUSSION

Bronchogenic cysts are congenital lesions derived from the primitive foregut, resulting from aberrant embryonic development involving portions of germinal tissue [1]. Depending on the airway and developmental stage involved, these cysts may manifest in various anatomical locations. Early-stage cysts are predominantly located in the mediastinum, whereas those forming later in the bronchial tree may arise within the lung parenchyma. Rarely, bronchogenic cysts may be observed in the neck, retroperitoneum, diaphragm, or other ectopic sites. Based on their anatomical localization, bronchogenic cysts are categorized into intrapulmonary, mediastinal, and ectopic types [2].

Clinical manifestations of intrapulmonary cysts are frequently complicated by infection and are characterized by symptomatic presentations. The severity of symptoms correlates with cyst size and the degree of compression exerted on adjacent structures. Small cysts are often asymptomatic, while larger cysts may cause cough, chest pain, dyspnea, or even superior vena cava obstruction syndrome. Sudden enlargement due to bleeding or secondary infection can lead to acute compressive or infective symptoms [3-5]. Conversely, mediastinal cysts are typically asymptomatic and are often identified incidentally during routine physical examinations. In the present case, the mediastinal type of bronchogenic cyst was associated with symptoms likely attributable to compression.

Currently, there are no specific diagnostic markers for bronchogenic cysts. Positive test results are usually related to associated comorbidities. Routine blood and biochemical tests are typically normal in asymptomatic cases. However, co-infection may result in elevated white blood cell and neutrophil counts, along with increased inflammatory markers, such as erythrocyte sedimentation rate and C-reactive protein. Intrapulmonary cysts are often lined with ciliated columnar epithelium and exhibit varying degrees of acute and chronic inflammation [6]. Tracheal compression or airway obstruction may result in blood gas abnormalities, such as reduced oxygen partial pressure and carbon dioxide retention.

Pathological examination reveals that bronchogenic cysts frequently lack direct communication with the bronchus, although they may exhibit a fibrous bronchogenic remnant. When a connection with the bronchus is present, the cyst may also contain gas. The cystic wall and bronchogenic wall are lined with pseudostratified ciliated columnar epithelium, although certain regions may be covered by either simple cuboidal or simple squamous epithelium. Additionally, the cyst wall typically consists of glands, cartilage, elastic fibers, and a small amount of smooth muscle. In cases of secondary infection, neutrophils and lymphocytes may infiltrate all layers of the cyst wall, resulting in the secretion of more viscous cystic fluid. This fluid is characterized by a high protein content and may also contain calcium-like deposits, mucoid material, or purulent components [7].

The differential diagnosis of mediastinal cysts is challenging due to the lack of distinctive features. Common imaging modalities for detecting bronchogenic cysts include chest X-ray and CT. Chest CT can accurately detect and localize lesions larger than 5 mm in the lungs, providing detailed information on the size, location, and relationship of the cyst to surrounding tissues [8]. Enhanced CT scans further assist in differential diagnosis and serve as a reference for preoperative planning. Approximately 8% to 23% of bronchogenic cysts are diagnosed through chest CT, which is considered the preferred imaging modality for this condition [9]. However, due to concerns regarding radiation exposure from CT scans, it is recommended that suspected cases initially undergo a combination of chest X-ray and targeted ultrasound as a feasible screening method prior to CT evaluation.

Ultrasound examination, as a simple and non-invasive procedure without radiation exposure, is commonly used in physical examinations, screening for congenital bronchogenic cysts in fetuses, and guiding procedures, such as thoracentesis. However, it is limited by the presence of intrapulmonary gas and ribs, allowing for the detection only of cysts located near the pleura without gas interference. Nonetheless, a broader application of echocardiography could significantly enhance the early detection of asymptomatic mediastinal bronchogenic cysts, as it enables systematic surveillance of the mediastinum during standard cardiac evaluations. In the present case, the preliminary diagnosis was made using ultrasound, underscoring its importance. Given the inherent limitations of pathological diagnosis via ultrasound, any mediastinal cyst detected through this modality should be assessed for various underlying pathologies.

Firstly, bronchogenic cysts are typically solitary, fluid-filled chambers that often appear anechoic on ultrasound. When the fluid contains high protein content, it can be differentiated from mediastinal lymph nodes. Secondly, cystic teratomas present as round or oval structures with smooth margins and a complete capsule, often displaying calcifications within the cystic wall. Thirdly, pericardial cysts are usually located in the right costodiaphragmatic angle, but they may also occur in the left costodiaphragmatic angle, valve region, or upper mediastinum. These cysts are generally asymptomatic, but they can cause pain upon torsion. On ultrasound, pericardial cysts appear as round or oval anechoic areas with thin, smooth walls that are non-communicative with the pericardial cavity and exhibit synchronous movement with cardiac cycles. Finally, esophageal cysts are predominantly located in the posterior and upper mediastinum and are visualized on ultrasound as round or oval anechoic structures with distinct outlines and smooth internal walls [10, 11].

To confirm pathological morphology, alleviate symptoms, prevent potential complications, or avoid misdiagnosis of malignant tumors, surgical resection of bronchogenic cysts is recommended [5, 12, 13]. Thoracotomy has traditionally been employed for this purpose, offering advantages, such as improved surgical exposure, lower equipment requirements, and higher rates of complete lesion resection [14]. In recent years, the Da Vinci robotic surgical system combined with thoracoscopic surgery has emerged as a viable approach for the resection of mediastinal cysts, including bronchogenic cysts. This technique offers advantages, such as minimal trauma, reduced impact on respiratory and circulatory function, rapid postoperative recovery, and fewer postoperative complications. However, its disadvantages include higher equipment and technical requirements, incomplete mass removal, and potential recurrence. The Da Vinci system is highly regarded for its three-dimensional vision, intelligent memory system, and precise operational capabilities [15].

Ultrasound- and CT-guided percutaneous cyst puncture and aspiration can aid in diagnosis while also alleviating space-occupying effects by extracting cystic fluid, particularly in larger cysts. This minimally invasive and cost-effective method has limitations, including increased risks of thoracic infections and recurrence [16]. For asymptomatic patients, those with mild symptoms, or individuals unable to tolerate surgery, medical management may be considered. Medical treatment is characterized by low risk, cost, and invasiveness, playing a critical role in symptom management and monitoring. Symptomatic management, such as anti-infective therapy for secondary infections and regular follow-up, is suitable for patients with small cysts, clear imaging diagnoses, low malignancy risk, or high surgical risk. A retrospective study reported that 45% of such patients eventually developed symptoms requiring surgical intervention [17].

## CONCLUSION

In summary, bronchogenic cysts are rare congenital respiratory anomalies, with chest pain and cough being common symptoms. The mediastinum is the most frequently affected location, followed by the lungs. CT is a key diagnostic tool, typically revealing round or oval thin-walled cystic lesions with regular margins. The cyst contents may include liquid, gas, or both. Pathological examination under microscopy demonstrates bronchial-specific tissues, including bronchial glands, cartilage, elastic fibers, and smooth muscle. The cyst lining consists of pseudostratified ciliated columnar epithelium or simple squamous epithelium. Surgical intervention is the primary treatment modality, with generally favorable postoperative outcomes.

## AUTHORS’ CONTRIBUTIONS

The authors confirm their contribution to the paper as follows: data collection: K.P.L.; analysis and interpretation of results: D.H.J.; drafting of the manuscript: L.Z. All authors have reviewed the results and approved the final version of the manuscript.

## Data Availability

The data and supporting information are included in the article.

## LIST OF ABBREVIATIONS

CT: Computed tomography
ECG: Electrocardiogram
RPA: Right pulmonary artery
LPA: Left pulmonary artery
DAO: Descending aorta

## STANDARDS OF REPORTING

CARE guidelines were followed.
